# Goodbye, idiopathic short stature: why “healthy short stature” is a necessary advancement

**DOI:** 10.20945/2359-4292-2026-0068

**Published:** 2026-06-25

**Authors:** Ivo J. Prado Arnhold, Berenice B. Mendonca

**Affiliations:** 1 Faculdade de Medicina da Universidade de São Paulo, São Paulo, SP, Brasil

IDIOPATHIC SHORT STATURE - A SHRINKING DIAGNOSIS APPLIED MOSTLY TO HEALTHY CHILDREN

We welcome the term “healthy short stature (HSS)” as it reduces the burden of a diagnosis to most children below the arbitrary definition of normal height ^(1)^. It also emphasizes that an individual might be healthy despite a genetic diagnosis ^(1)^.

Idiopathic short stature (ISS) has been defined as a condition in which the height of an individual, with normal birthweight and GH sufficient, is more than 2 SD score (SDS) below the corresponding mean height for a given age, sex, and population group, without evidence of systemic, endocrine, nutritional, or chromosomal abnormalities ^(2)^.

As technology and our knowledge progresses, we need to adapt and improve nomenclatures and management.

Assuming that height has a Gaussian distribution, 2.3% of the normal population will have a height below -2.0 SD. We consider this threshold (-2 SD) as an indication for investigation to exclude manageable conditions but not as a threshold for treatment ^(3)^.

Common sense suggests that the farther the individual is from the mean, or from the parental height, the more likely it is due to a disease, more likely that a genetic abnormality can be found ^(4)^, and the more intense the search for a cause should be attempted. Also more likely is the burden of short stature, physically and psychosocially ^(5)^. Conversely, the closer to -2.0 SD, the higher the likelihood that a healthy child is a variation of normal.

In the US, according to the FDA, not all children with ISS are granted GH therapy, but only those with a height below -2.25 SD (1.2%). This excludes those between centiles 1.2 to 2.25 (approx. 45%) of patients labelled as ISS. Depending on the availability of rhGH, lower height limits of height may warrant treatment with growth promoting agents ^(2)^.

It is important to stress that idiopathic SS as well as healthy SS as suggested by Jorge et al, are diagnoses of exclusion. In fact, the authors expand the list of conditions that should be excluded in order to make the diagnosis of healthy short stature ^(1)^.

The authors refer to the lack of standardization for the measurement of body proportions. Interestingly, the sitting height to standing height SDS (SH/H) is a useful parameter which can be applied to different populations independently from the patient’s height. For example, the standards developed for the Dutch population can be applied to Brazilian children ^(6,7)^. Sitting height can be measured in a specifically designed device, but in practice, it is enough to sit the patient on a removable box (usually 60 cm high) under the same calibrated stadiometer for height measurement and subtracting the height of the box ^(7)^.

Genetic testing shows importance of thorough clinical examination, as it is now not infrequent that after the finding of a genetic alteration the patient’s phenotype is revisited and minor or previously undetected abnormalities are found.

An important and very frequent condition with an acceptable height prognosis that can be considered as healthy short stature is the constitutional delay in growth and puberty (CDGP) ^(8)^. Superimposed to the normal variability in height, there is the variability on the age of development of puberty. Because growth velocity increases significantly during the pubertal growth spurt, differences in the timing of puberty can result in important height differences among peers of the same age. In the 2008 consensus on ISS, CDGP was considered a sub-classification of ISS ^(2)^. Before puberty, family history and a delayed bone age are reassuring but CDGP remains a diagnosis of exclusion made after puberty. The genetic basis of delayed puberty is being studied ^(9-12)^.

The development and widespread use of genetic testing also imposes new challenges: the discovery of variants classified as of uncertain significance (VUS) necessitate further studies to determine its real participation in the phenotype. The discovery of pathogenic variants in normal individuals indicate the possible variable penetrance and expressivity of these conditions ^(13)^.

The authors acknowledge that “Increasing understanding of the monogenic and polygenic architecture of short stature, alongside with genotype-specific treatment responses, will facilitate a transition toward more precise, genetically informed treatment protocols” ^(1)^.

The further development and improvement of polygenic scores (PGS) for both height and pubertal timing for all populations will likely help distinguish individuals who have inherited a polygenic variation of height and/or puberty from those in whom another diagnosis should be searched for.

After careful evaluation, we believe that the term healthy short stature is a designation that most short children deserve and includes height among the many individual characteristics.

In conclusion, the article by Jorge and colleagues is highly timely ^(1)^. Pediatric endocrinology has a role to play in abandoning terms that do not reflect current knowledge. We recommend the progressive adoption of the term “Healthy Short Stature” in clinical practice, scientific publications, and health systems. Considering that an increase in height represents more a desire than a need, physicians should emphasize the normality and qualities of HSS children, rejecting the concept that being taller means being better.

## Data Availability

datasets related to this article will be avail-able upon request to the corresponding author.
